# Thoracolumbar Posterior Pedicle Screw Fixation and Laminectomy for an Osteoporotic Vertebral Fracture Resulting From Acute Lymphoblastic Leukemia in an Adult Patient: A Case Report

**DOI:** 10.7759/cureus.73710

**Published:** 2024-11-14

**Authors:** Yusuke Ota, Takayuki Awaya, Yoshitaka Nagashima, Toshiki Fukuoka, Osamu Suzuki, Yusuke Nishimura

**Affiliations:** 1 Department of Neurosurgery, Nagoya Ekisaikai Hospital, Nagoya, JPN; 2 Department of Neurosurgery, Nagoya University Graduate School of Medicine, Nagoya, JPN; 3 Department of Neurological Surgery, Nagoya University Hospital, Nagoya, JPN

**Keywords:** acute lymphoblastic leukemia (all), osteoporotic vertebral body fracture (ovf), posterior decompression and fixation, secondary osteoporosis, thoracolumbar spine

## Abstract

Vertebral fractures (VFs) occasionally appear as the first manifestation of acute lymphocytic leukemia (ALL) in children. However, in adults, it is uncommon for VFs to lead to a diagnosis of ALL, and surgical intervention is even rarer. We encountered a case of a 42-year-old man with ALL who presented with acute severe back pain, lower limb numbness, dysuria, and hamstring weakness. A CT scan revealed a burst fracture of the L2 vertebra with spinal canal stenosis. Laboratory results indicated pancytopenia, and an emergency bone marrow biopsy suggested leukemia. Emergency surgery, including lumbar laminectomy (L1-L3) and pedicle screw fixation (T11-L4), was performed, after which the patient’s back pain and dysuria resolved, and hamstring strength improved. Bone mineral density testing revealed osteoporosis, later diagnosed as secondary to ALL. The patient was subsequently diagnosed with ALL with Philadelphia chromosome abnormality and underwent chemotherapy and bone marrow transplantation. He remains in remission four years after the operation. In cases of symptomatic nerve compression due to VFs secondary to adult-onset ALL, early posterior decompression and fixation can help maintain good performance status and lead to favorable outcomes. This case represents the first reported instance where surgical intervention was performed specifically to maintain performance status for early chemotherapy in such patients. Additionally, in young adults presenting with osteoporotic VFs, hematologic malignancies such as ALL should be considered in the differential diagnosis.

## Introduction

Acute lymphoblastic leukemia (ALL) is a malignant hematologic disorder characterized by the uncontrolled proliferation of immature lymphoid cells in the bone marrow, blood, and other organs. ALL is the most common malignancy in children, with almost 80% of cases occurring in childhood and only about 20% in adulthood [1]. Compared to children, adult patients generally have a poorer prognosis [2]. ALL typically presents with non-specific symptoms such as fatigue, prolonged fever, weight loss, night sweats, and swollen lymph nodes, along with signs of bone marrow failure (anemia, thrombocytopenia, leukopenia) [2]. Vertebral fractures (VFs) can occasionally appear in children as the first manifestation of ALL, with the incidence of VFs reported to be more than 2,000 times higher than in the general pediatric population [3,4]. In children, fractured vertebral bodies can remodel after ALL treatment [4]. Some reports have described cases of ALL with spinal cord compression caused by tumor masses, which have been successfully treated with chemotherapy and radiotherapy, often achieving good outcomes without the need for surgical intervention [5,6].

This case report details a middle-aged patient who developed osteoporotic VFs secondary to ALL, requiring spinal decompression and fusion surgery. To our knowledge, this is the first report of surgical intervention for an adult patient with ALL-induced osteoporotic VFs.

## Case presentation

**Figure 1:**
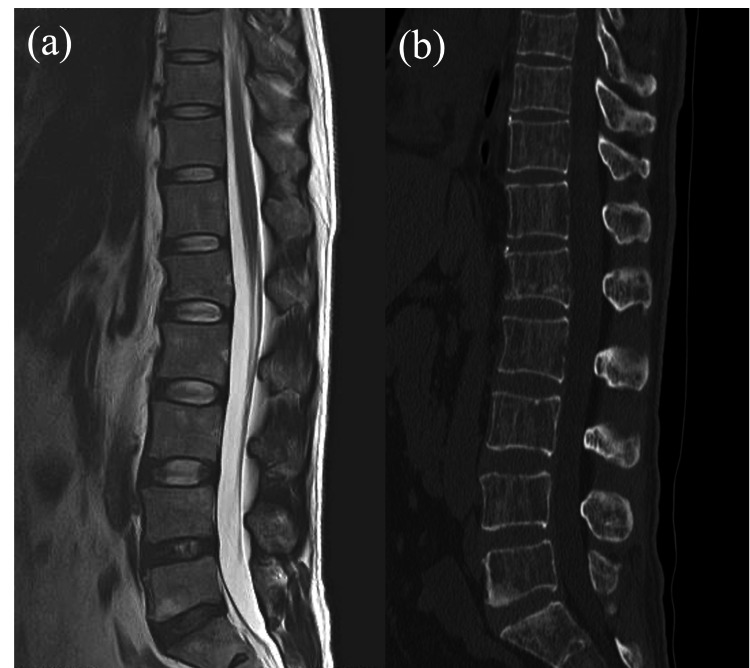
Initial MRI and CT scan. Initial MRI (a) and CT scan (b) showing no obvious L2 vertebral fracture. A slight deformation at L1 was observed on CT, with no signal change on MRI, suggesting an old fracture at L1.

A 42-year-old man with no significant medical history experienced chronic back pain, which led to several visits to the emergency department. Initial CT scans revealed urolithiasis but no acute VFs, and slight deformation suggestive of an old fracture at L1 was observed on MRI and CT (Figure 1).

**Figure 2:**
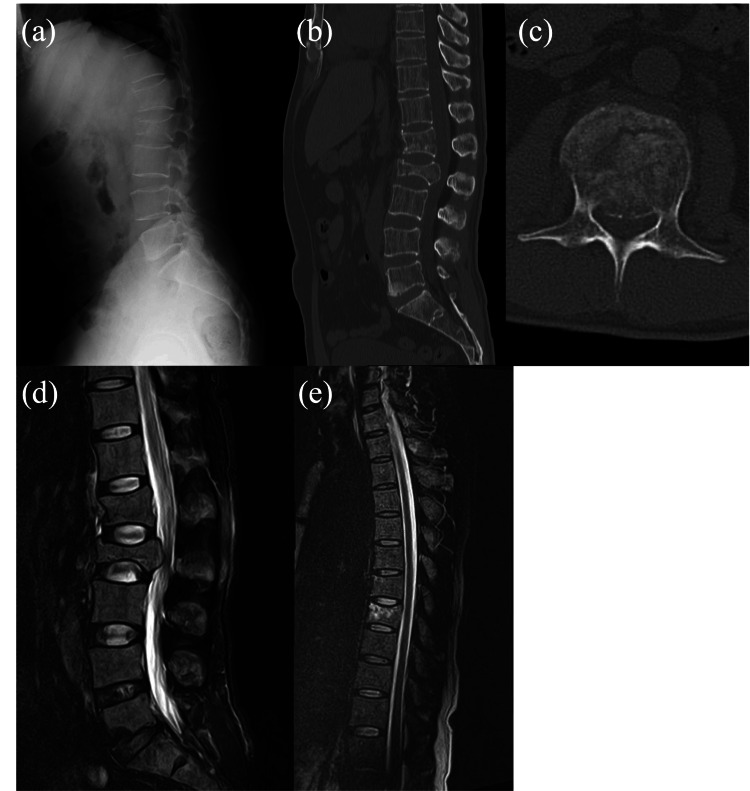
X-ray, CT scan, and MRI on the day of admission. Imaging from the day of admission. (a) X-ray of the thoracolumbar spine. (b, c) CT scan showing axial and sagittal views with severe spinal canal stenosis caused by an L2 burst fracture and an L1 compression fracture. (d, e) MRI of the thoracic spine with a T2-weighted image showing signs of T7 compression fracture.

**Figure 3:**
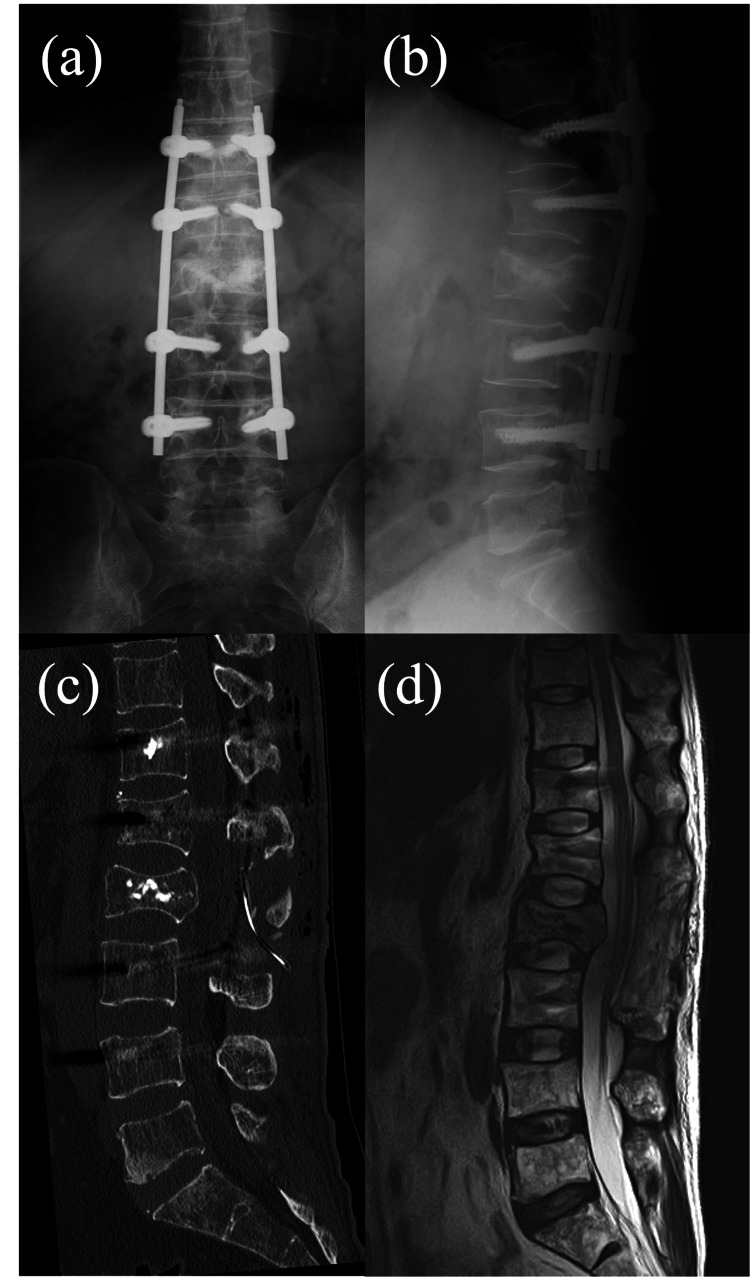
Postoperative images. (a, b) Postoperative X-rays of the thoracolumbar spine. (c, d) Sagittal CT and MRI T2-weighted imaging.

However, the patient returned with acute severe back pain, lower limb numbness, dysuria, and hamstring weakness, though he remained ambulatory. A repeat X-ray, CT scan, and MRI revealed a burst fracture of the L2 vertebra and spinal canal stenosis at the same level (Figure 2). A whole-body CT revealed no abnormalities beyond the vertebral fracture. Laboratory data revealed pancytopenia, with a white blood cell count of 2,600/µL, hemoglobin of 11.5 g/dL, and platelet count of 43,000/µL. In biochemical tests, there were no significant abnormalities. An emergency bone marrow biopsy suggested leukemia (Figure 3). Thus, it was determined that the VF was caused by secondary osteoporosis resulting from leukemia.

**Figure 4:**
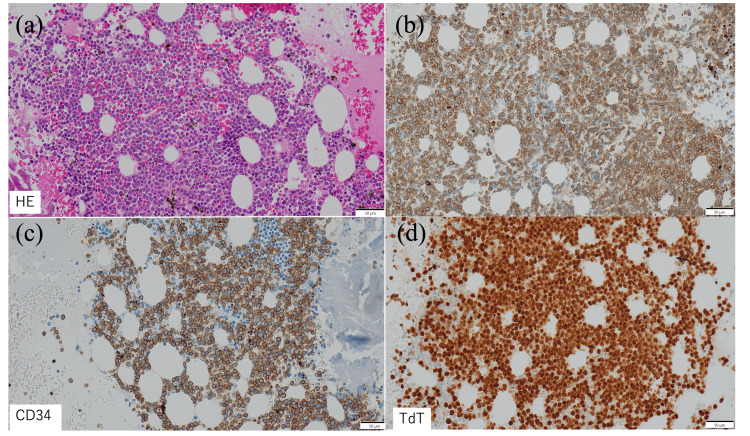
Pathological results of bone marrow examination. (a) Hematoxylin and eosin-stained image showing small, uniformly sized cells densely infiltrating. (b) CD79a-positive B-lymphocyte cells, (c) CD34-positive blasts, and (d) TdT-positive cells, confirming the diagnosis of lymphoblastic leukemia/lymphoma (all images were captured at 400× magnification).

The patient underwent emergency surgery for symptomatic nerve compression caused by the L2 burst fracture. A lumbar laminectomy of L1-L3 and pedicle screw fixation from T11 to L4 were performed (Figure 4). Postoperatively, his back pain and dysuria resolved, and his hamstring strength improved.

Further postoperative examination, including bone mineral density measurement, indicated osteoporosis (63% of the young adult mean <-2.5 SD). MRI revealed additional compression fractures at T7 and L1, confirming the diagnosis of secondary osteoporosis. The patient was diagnosed with Philadelphia chromosome-positive ALL. Despite the challenges, the patient tolerated postoperative chemotherapy and bone marrow transplantation well and has remained in remission for four years following surgery.

No identifying information is included in this case report to protect patient privacy. Written informed consent for publication was obtained from the patient. Additionally, this study was conducted in accordance with institutional ethical standards and received approval from our Institutional Review Board.

## Discussion

VFs related to ALL are well documented in pediatric populations, where the incidence is significantly higher than in the general population [4,7]. Osteoporosis and VFs can occur at any time during ALL, often exacerbated by chemotherapy [8]. In children, the fractured vertebral bodies can remodel following treatment [4]. However, in adults, VFs before diagnosis are rare, and adult patients present distinct clinical challenges [9].

In adults, determining the appropriate treatment is complicated by the need to preserve performance status for subsequent chemotherapy. Conservative treatments may not be adequate in cases where neurological deficits are present, such as in our patient, who exhibited vesicorectal dysfunction and lower limb weakness. In such cases, early surgical intervention to preserve neurological function and maintain performance status is essential.

Most reports of spinal cord dysfunction in ALL are related to spinal compression caused by tumor masses rather than VFs [5,6]. In these cases, chemotherapy and radiotherapy are typically sufficient to manage the symptoms before permanent neurological damage occurs. However, when spinal cord compression results from VFs, especially unstable fractures such as the burst fracture in our patient, surgical intervention is necessary.

This case represents the first report of surgical intervention for maintaining performance status to enable early chemotherapy in a middle-aged adult with ALL-related osteoporotic VFs and neurological impairment. Early recognition of the potential hematologic malignancy was critical in ensuring timely treatment and favorable patient outcomes.

Given the rarity of osteoporotic VFs in young adults, this case highlights the need for clinicians to consider hematologic malignancies, such as leukemia, when encountering such fractures, particularly in patients with no history of trauma. Early diagnosis and intervention in these cases are crucial for improving patient outcomes and ensuring the timely initiation of necessary therapies, such as chemotherapy.

This case report has several limitations. First, as a single case report, the findings may not be generalizable to other patients with ALL. Further case accumulation and research are needed to determine whether the same approach would be effective in similar cases. Second, although remission was confirmed with a four-year follow-up, longer-term follow-up data is lacking. Consequently, information on spinal stability and the risk of recurrence after surgery remains limited.

## Conclusions

In adults with VFs secondary to ALL, early posterior decompression and fixation can preserve neurological function and maintain performance status, allowing for the timely initiation of chemotherapy. Notably, this case represents the first report of surgical intervention aimed at preserving performance status to facilitate early chemotherapy for an adult patient with ALL-induced osteoporotic VFs and neurological impairment.

This case underscores the importance of considering hematologic malignancies, such as leukemia, in the differential diagnosis when encountering VFs in younger adults, especially in cases of osteoporosis. Prompt recognition and appropriate intervention in such cases are essential to ensure optimal outcomes, particularly when early initiation of cancer therapy is critical.
